# An Investigation of the Significance of Residual Confounding Effect

**DOI:** 10.1155/2014/658056

**Published:** 2014-02-17

**Authors:** Wenbin Liang, Yuejen Zhao, Andy H. Lee

**Affiliations:** ^1^National Drug Research Institute, Curtin University, G.P.O. Box U 1987, Perth, WA 6845, Australia; ^2^Northern Territory Department of Health, Darwin, NT 0800, Australia; ^3^School of Public Health, Curtin University, Perth, WA 6845, Australia

## Abstract

*Background*. Observational studies are commonly conducted in health research. However, due to their lack of randomization, the estimated associations between the outcome and the exposure can be affected by unmeasured confounding factors. It is important to determine how likely a significant association observed between an outcome variable and a noncausally related exposure may be introduced by residual confounding factors. *Methods*. A simulation approach is developed based on the sufficient cause model to test the likelihood of significant associations observed between a noncausally related exposure and the outcome. *Results*. Based on the estimates from all 500 replicates, the association between the exposure and the outcome is found to be significant in 386 (77%) replicates when all confounders (component causes) are controlled for in the model. However, when a subset of real component causes and some noncausal factors are controlled for in the model, the association between exposure and the outcome becomes significant in 487 (97%) replicates. *Conclusion*. Even when all confounding factors are known and controlled for using conventional multivariate analysis, the observed association between exposure and outcome can still be dominated by residual confounding effects. Therefore, an observed significant association apparently provides limited evidence for a causal relationship.

## 1. Introduction

Ethical and budgetary constraints often limit the application of experimental study designs in health research, so that observational studies such as cohort or case-control studies have been widely undertaken as methodological alternatives [1–5]. However, due to the lack of randomization, the estimates so obtained can be influenced by uncontrolled or unmeasured confounders and typically, the confounders bias estimates from their true values [6–12]. According to the epidemiological literature, a confounder must meet the following conditions: (i) being a cause of the disease, or a proxy of cause(s), in unexposed people; (ii) being correlated with exposure in the study population; (iii) not being an intermediate step in the causal pathway between the exposure and the disease [1, 13–16]. To deal with confounding effects, known or suspected confounders are measured together with the exposure and outcome of interest. Multivariate analyses are then performed to measure the association between the exposure and the outcome while attempting to remove the effects of such known or suspected confounders [8, 13, 17–19].

Under the sufficient cause model, a sufficient cause means a complete causal mechanism, which can be defined as a combination of minimal conditions (necessary elements) and events that inevitably produce disease, while the necessary elements that constitute a sufficient cause are component causes [2]. It is common that component causes and compositions of sufficient causes are unknown, with simultaneous existence of measurement errors, misclassifications for exposures, confounders, and outcomes [8, 20–23]. Consequently, the estimated associations between the outcome and the exposure remain likely to be affected by unmeasured confounding factors. For example, even in well-designed studies, significant protective associations occurred between true nonprotective exposures and outcomes are actually caused by unmeasured confounding factors [24, 25]. It is thus important to investigate how likely a significant association observed between an outcome variable and a noncausally related exposure may be introduced by residual confounding factors. In this study, we develop a simulation approach to test the likelihood of observing significant associations between a noncausally related exposure and the outcome variable based on standard multivariate analysis, given that the compositions of sufficient causes are not recognized, but either all risk factors/component causes are known and controlled, or only some of the risk factors/component causes are known and controlled. There are two objectives: (1) to investigate the likelihood of false positive observations in observational studies, (2) to propose a simulation framework for assessing epidemiologic methods which deal with confounding effects.

## 2. Methods

### 2.1. Overview of the Simulation

The simulation process follows the sufficient cause model [2]. For an event to occur, at least one sufficient cause has to occur. The components of a sufficient cause are randomly chosen from a pool of low to moderate correlated variables, which include the exposure of interest and 99 other variables. The exposure of interest is set to be *non*causal for the outcome and therefore it will never be chosen as a component for a sufficient cause. Given the correlation among the 100 variables, every chosen variable is a potential confounding factor for the association between the exposure and the outcome. The association between the exposure and the outcome is then estimated using a logistic regression model, while controlling for (i) all component causes; and (ii) some of the component causes (selected at random). The simulations contain 500 replicates, with each replicate being generated through an independent process. All simulations are performed using the STATA package release 12. The procedures involved in each replicate are outlined below. Details of the simulation procedure, including the sufficient cause model and the estimation process, are provided in the Appendix.

(1) Generate a pool of low to moderate correlated random variables from the uniform [0,1) distribution: *T*_100×50000_ = {*T*_*i*,*n*_}, *i* = (1,2, 3,…, 100), *n* = (1,2, 3,…, 50000).
(2) Determine the composition of sufficient causes and the threshold values of components. The total number for the types of sufficient causes for *Y* is randomly chosen from (1,2, 3,…, 9). Components for each type of sufficient causes are randomly selected from *T*_*i*,*n*_, *i* = (2,3,…, 100). *T*_1_ is taken as the exposure, which is set to be noncausal for *Y*. For each observation, a sufficient cause is set to occur, when each of its components has a value higher than its specific threshold value. The threshold value is specific for each component as well as each type of sufficient cause, and it is randomly chosen from a uniform [0.5, 0.9) distribution. This allows the threshold values to vary between components as well as between different sufficient causes for the same component. To reflect the fact that exact threshold values are typically unknown, *T*_*i*,*n*_ are then dichotomized into binary form denoted by *X*_*i*,*n*_, *i* = (1,2, 3,…, 100), *n* = (1,2, 3,…, 50000), by applying the following rule: *X*_*i*,*n*_ is set to 1 if *T*_*i*,*n*_ > 0.7, and 0 otherwise. Here, the mean 0.7 of a uniform [0.5, 0.9) variable is used instead of applying the exact threshold values, in order to account for unavoidable measurement errors and misclassifications in confounders and exposures.
(3) Generate competing events for *Y*, *E*_*n*_, *n* = (1,2, 3,…, 50000). Note that *E* is independent of *T* and *X*.
(4) Generate small random errors for *Y* to represent measurement errors of outcome and to smooth the computing process. *Q* is a Bernoulli distributed random variable, being independent of *E* and *X* and only accounts for a small proportion of variance of *Y*.
(5) Determine the status (occur or not occur) of *Y*.
(6) Determine the known (not necessary the fact) causal factors for *Y* through a random process.
 Details of steps  1 to 6 can be found in the Appendix.
(7) Estimate the effect of *X*_1_ on *Y* when all component causes are identified. There is no noncausal factor being mistaken as causal factor. We have
  (1) $$\begin{array}{c} P\left(Y_{n}=1\;|\;X_{i,n},C\right)=\frac{\exp\left(\beta_{1}X_{1,n}+\sum_{i=2}^{100}\beta_{i}X_{i,n}C_{i}\right)}{1+\exp\left(\beta_{1}X_{1,n}+\sum_{i=2}^{100}\beta_{i}X_{i,n}C_{i}\right)}, \end{array}$$

where *C*_*i*_ indicates whether *X*_*i*_ is involved in at least one sufficient cause of *Y*, that is, *C*_*i*_ = 1 if true and *C*_*i*_ = 0 otherwise. Here, *β*_1_ and *β*_*i*_ are the estimated effects of *X*_1_ and each of the component causes on *Y*, respectively. To estimate the effect of *X*_1_ on *Y* when only some component causes are known, and there are some noncausal factors being mistaken as causal factors, we have

(2) $$\begin{array}{c} P\left(Y_{n}=1\;|\;X_{i,n},K\right)=\frac{\exp\left(\beta_{1}^{\prime }X_{1,n}+\sum_{i=2}^{100}\beta_{i}^{\prime }X_{i,n}K_{i}\right)}{1+\exp\left(\beta_{1}^{\prime }X_{1,n}+\sum_{i=2}^{100}\beta_{i}^{\prime }X_{i,n}K_{i}\right)}, \end{array}$$

where *K*_*i*_ indicates whether *X*_*i*_ is “known” or suspected to be involved in at least one sufficient cause of *Y*, *β*_1_′, and *β*_*i*_′ are the estimated effects of *X*_1_ and each of the “known” risk factors on *Y*, respectively.

## 3. Results

Data obtained from replicate 1 is used as an example.  Table 1 shows details of the sufficient causes and their components for replicate 1. Overall, the incidence rate (per 1000 observation units) for *Y* is 32.4, while it is 20.2 among unexposed observations (*X*_1_ = 0) and 89.0 among exposed observations (*X*_1_ = 1). This leads to an observed crude exposed-to-unexposed risk ratio of 4.4, though the exposure is not causal for *Y*. Moreover, as shown in Table 2, the strength of association between exposure and confounders is considerably low, with low level of misclassifications for confounder status. Given that all confounding factors (component causes) are controlled for in the model, the effect of exposure remained significant (*P* < 0.001).  Table 3 suggests that the effect of exposure is further biased away from the null when only a subset of real component causes and some noncausal factors are controlled in the model.

Based on the estimates from all replicates, the association between the exposure and the outcome *Y* is found to be significant in 386 (77%) out of the 500 replicates when all confounders (component causes) are controlled in the model. However, when a subset (rather than all) of real component causes and some noncausal factors are controlled in the model, the association between the exposure and the outcome *Y* becomes significant in 487 (97%) out of the 500 replicates.

**Figure 1:**
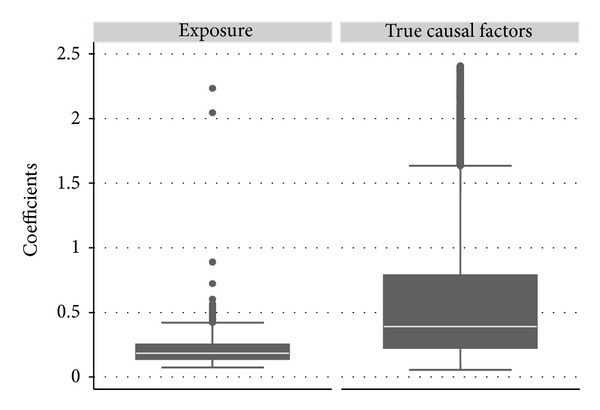
Difference in estimate distributions between the exposure and the real component causes. The upper and lower adjacent lines indicate the upper and lower adjacent values, respectively; the upper and lower edges of the boxes indicate 75th percentiles and 25th percentiles, respectively; and the white lines in the boxes indicate the medians. (The upper limit of the graph is set to 2.409, the 95th percentile for coefficients of the real component causes).

In addition, Figure 1 indicates that when adjusting for all the real component causes, the significantly estimated effect of the exposure is on average substantially smaller than the effects of real component causes. The mean (standard deviation), 25th, 50th, and 75th percentiles of the significant coefficients (natural logarithm of the odds ratio) are 0.22 (0.17), 0.14, 0.18, and 0.25, respectively for the noncausal exposure and are 0.73 (0.79), 0.23, 0.42, and 0.927, respectively, for the real component causes.

## 4. Discussion

In observational studies, when a statistical significant association arises between an exposure and the outcome in the multivariate analysis, it is usually considered as supportive evidence for causal relationship [8]. We adopt the sufficient cause model in the simulation process to investigate how likely a significant association between the exposure and the outcome may be observed when there is no causal association between the two in an observational study setting. The results indicate that significant associations between the exposure and its noncausal related outcomes are presented in more than 70% of the situations, even when assuming that all confounders (causal factors) are known to researchers and controlled for in the multivariate analysis. In reality, many component causes of a disease are unknown [8, 20–23].

Moreover, results from the simulation study suggest that under the conventional multivariate analysis approach, residual confounding effects remain strong enough to influence the observed associations and an observed significant association provides only limited evidence for a causal relationship. Therefore, new methods are required to handle residual confounding effects. The simulation design adopted in this study can also serve as a platform to evaluate the performance of such methods.

There are several advantages of our simulation design. Firstly, although all component causes and sufficient causes are determined through random process, they are all tracked and measured, unlike collected data where most pieces of information on component causes and sufficient causes are unknown and unmeasurable. Secondly, for specific exposures and outcomes, information from existing literature can be easily adopted into the simulation design. Thirdly, the simulation design can be adjusted to fit specific prior assumptions on the distributions and correlations among component causes and the exposure as well as compositions of sufficient causes. Hence it is possible to obtain estimates on the effects of the exposure under different prior assumptions.

## 5. Conclusion

This study demonstrates that even when all confounding factors are known and controlled for using conventional multivariate analysis, the observed association between exposure and outcome can still be dominated by residual confounding effects. An observed significant association apparently provides limited evidence for a causal relationship.

## Figures and Tables

**Table 1:** Sufficient causes and their components for replicate 1.

Type of sufficient cause	Components (cut-off points)	Observed frequency for the 50,000 observations
A	*X*_17_ (0.847), *X*_50_ (0.850)	421
B	*X*_7_ (0.521), *X*_29_ (0.881), *X*_53_ (0.619)	515
C	*X*_18_ (0.754), *X*_20_ (0.626), *X*_21_ (0.504), *X*_38_ (0.642), *X*_91_ (0.617)	741

As described in the simulation design and the appendix, the total number of sufficient causes and the components of each possible sufficient cause vary between replicates and are determined by independent random process (i.e., sufficient cause A has two components: *X*_17_ and *X*_50_; sufficient cause B has three components: *X*_7_, *X*_29_, and *X*_53_).

**Table 2:** Source and magnitude of bias in replicate 1.

Confounder/ Component	Correlation with exposure^1^	Percentage of misclassification^2^
*X*_17_	0.183	13.4%
*X*_50_	0.160	14.4%
*X*_7_	0.135	26.7%
*X*_29_	0.150	15.5%
*X*_53_	0.181	11.6%
*X*_18_	0.227	5.89%
*X*_20_	0.155	10.8%
*X*_21_	0.292	31.2%
*X*_38_	0.188	7.9%
*X*_91_	0.282	11.4%

^1^Measured as the correlation coefficient between binary form of component (occurred or not occurred) and binary from of exposure in the 50,000 observations for replicate 1.

^2^Measured as 1 minus the proportion of correct classification of confounder/component status (occurred or not occurred) in the 50,000 observations for replicate 1.

**Table 3:** Estimates from multivariate analysis in replicate 1.

	Model adjusted for all component causes			Model adjusted for randomly selected component causes and noncausal factors		
	Odds ratios	95% Confidence interval		Odds ratios	95% Confidence interval	
(*X*_1_) Exposure	1.31	1.17	1.48	1.71	1.52	1.92
*X*_5_	—			1.48	1.32	1.65
*X*_7_	1.61	1.44	1.81	—		
*X*_11_	—			1.95	1.73	2.20
*X*_14_	—			1.69	1.50	1.89
*X*_17_	2.45	2.18	2.75	—		
*X*_18_	4.55	4.03	5.13	—		
*X*_20_	2.67	2.38	2.99	—		
*X*_21_	1.49	1.32	1.67	1.93	1.71	2.17
*X*_23_	—			1.47	1.31	1.65
*X*_29_	2.68	2.38	3.01			
*X*_32_	—			1.40	1.26	1.57
*X*_37_	—			1.41	1.26	1.57
*X*_38_	3.10	2.76	3.48	—		
*X*_50_	2.41	2.16	2.70	—		
*X*_53_	1.90	1.69	2.13	2.12	1.90	2.37
*X*_57_	—			2.06	1.83	2.32
*X*_69_	—			1.39	1.25	1.56
*X*_90_	—			1.17	1.04	1.31
*X*_91_	2.28	2.03	2.56	—		

—: variables not included in the model.

## Appendix

### Details of Steps 1 to 6 in Simulation Procedure

1. Generate a matrix of correlated random variables, *T*_100×50000_ = {*T*_*i*,*n*_}, its corresponding matrix *G*_9×100×50000_ = {*G*_*j*,*i*,*n*_}, and *X*_100×50000_ = {*X*_*i*,*n*_}, where *j* = (1,2, 3,…, 9), *i* = (1,2, 3,…, 100), and *n* = (1,2, 3,…, 50000). *G*_*j*,*i*,*n*_ indicates whether *T*_*i*,*n*_ passes its threshold value and becomes active or occurs in the *j*th sufficient cause (if it is a component of the *j*th sufficient cause) of the *n*th observation. *X*_*i*,*n*_ is a proximate measure of *G*_*j*,*i*,*n*_ given that the exact threshold value is usually unknown.
  - *T*_*i*,*n*_ = *V*_*in*_ *P*_*i*_ + *U*_*n*_(1 − *P*_*i*_) is a linear combination of a variable component (*V*_*i*_) and a unique component (*U*) for each *i*, with both being uniform [0,1) distributed random variables, and *P*_*i*_ is a random proportion drawn from a uniform [0.3, 0.6) distribution. The range [0.3, 0.6) is chosen in order to set a low to moderate level of correlation among *T*. The mean (standard deviation), 25th, 50th, and 75th percentiles of the correlation coefficients for the matrix *T* are 0.35 (0.13), 0.25, 0.33, and 0.45, respectively.
  - *G*_*j*,*i*,*n*_ is set to 1 if *T*_*i*,*n*_ > *A*_*j*,*i*_ and 0 otherwise, where *A*_*j*,*i*_ takes on a random value drawn from an uniform [0.5, 0.9) distribution. The mean (standard deviation), 25th, 50th, and 75th percentiles of the correlation coefficients for the matrix *G* are 0.16 (0.07), 0.11, 0.15, and 0.20, respectively.
  - *X*_*i*,*n*_ is set to 1 if *T*_*i*,*n*_ > 0.7 and 0 otherwise, where 0.7 is the expected value of the uniform [0.5, 0.9) distribution. The mean (standard deviation), 25th, 50th, and 75th percentiles of the correlation coefficients for the matrix *X* are 0.19 (0.07), 0.13, 0.17, and 0.24, respectively.
2. Determine sufficient cause compositions and their components.
  - Components for nine possible sufficient causes for *Y* are determined. Let *C*_*j*,*i*_, *j* = (1,2, 3 …, 9), *i* = (1,2, 3,…, 100) indicate whether *T*_*i*_ is a component of the *j*th possible sufficient cause: if *T*_*i*_ is component of the *j*th possible sufficient cause, *C*_*j*,*i*_ = 1, and 0 otherwise. *C*_*j*,*i*_ takes on a random value drawn from the Bernoulli distribution with probability of success *H*_*i*_, which is derived (rescaled) from a gamma distribution with both shape parameter and scale parameter equal to 1. For each sufficient cause if the components are less than 2, that is, for a given *j* if ∑_*i*_ *C*_*i*_ < 2, then all components are redetermined through the same random process.
  - Determine whether a possible sufficient cause occurs. Let *O*_*j*,*n*_ = 1 when all components for the *j*th possible sufficient cause become active or occur in the *n*th observation; that is, ∑_*i*_ *G*_*j*,*i*,*n*_ *C*_*j*,*i*_ = ∑_*i*_ *C*_*j*,*i*_; otherwise *O*_*j*,*n*_ = 0, *i* = (2,3,…, 100), *j* = (1,2,…, 9), and *n* = (1,2, 3,…, 50000).
  - Choose real sufficient causes from the nine possible sufficient causes. Let *F*_*j*_, *j* = (1,2,…, 9) denote whether the *j*th possible sufficient cause is a real sufficient cause for *Y*. If the *j*th possible sufficient cause is a real sufficient cause, then *F*_*j*_ = 1 and 0 otherwise. *F*_*j*_ takes on a random value drawn from the Bernoulli distribution with probability of success 0.5. If there is no real sufficient cause assigned, that is, ∑_*j*_ *F*_*j*_ < 1, then the real sufficient causes for *Y* are redetermined through the same random process.
3. Determine competing events. Let *E*_*n*_ denote the competing events for outcome *Y*, *n* = (1,2, 3,…, 50000). *E* is a Bernoulli distributed random variable with a probability of success 0.001, value of success (competing events occurred) being 1, and value of failure (competing events not occurred) being 0. *E* is independent of *X*.
4. Determine small random errors for *Y*. Let *Q*_*n*_ denote a small random error of *Y*, *n* = (1,2, 3,…, 50000). *Q* is a Bernoulli distributed random variable with a probability of success 0.001, value of success being 1, and value of failure being 0. *Q* is independent of both *E* and *X*.
5. Determine the status of outcome *Y*. Let *Y*_*n*_ = {0,1}, *n* = (1,2, 3,…, 50000) denote the outcome not occurred or occurred, respectively. Value of each *Y*_*n*_ is determined as follows. For each observation *n*, *Y*_*n*_ = 1 (outcome occurred) if *Q*_*n*_ = 1, or for *j* = (1,2, 3 …, 9), ∑_*j*_ *O*_*j*,*n*_ *F*_*j*,*n*_ ≥ 1 when *E*_*j*,*n*_ = 0; otherwise *Y*_*j*,*n*_ = 0 (outcome not occurred).
6. Determine the known/suspected causal factors, in other words, potential confounding factors. Let *K*_*i*_, *i* = (2,3, 4 …, 100)  denote the researcher's knowledge (not necessary the fact) on *X*_*i*_ in relation to its confounding effect on the association between *X*_1_ and *Y*. *K*_*i*_ is a random value drawn from the Bernoulli distribution with a probability of success 0.1 + ∑_*j*_ *C*_*j*,*i*_ *F*_*j*_/10, value of success being 1, and value of failure being 0. ∑_*j*_ *C*_*j*,*i*_ *F*_*j*_ is the total number of real sufficient causes that included *X*_*i*_ as a component.
